# An Approach for Assessing Human Health Vulnerability and Public Health Interventions to Adapt to Climate Change

**DOI:** 10.1289/ehp.8430

**Published:** 2006-07-11

**Authors:** Kristie L. Ebi, R. Sari Kovats, Bettina Menne

**Affiliations:** 1 ESS, LLC, Alexandria, Virginia, USA; 2 Department of Public Health and Policy, London School of Hygiene and Tropical Medicine, London, United Kingdom; 3 World Health Organization Regional Office for Europe, European Centre for Environment and Health, Rome, Italy

**Keywords:** adaptation, climate change, climate variability, human health methods, vulnerability

## Abstract

Assessments of the potential human health impacts of climate change are needed to inform the development of adaptation strategies, policies, and measures to lessen projected adverse impacts. We developed methods for country-level assessments to help policy makers make evidence-based decisions to increase resilience to current and future climates, and to provide information for national communications to the United Nations Framework Convention on Climate Change. The steps in an assessment should include the following: *a*) determine the scope of the assessment; *b*) describe the current distribution and burden of climate-sensitive health determinants and outcomes; *c*) identify and describe current strategies, policies, and measures designed to reduce the burden of climate-sensitive health determinants and outcomes; *d*) review the health implications of the potential impacts of climate variability and change in other sectors; *e*) estimate the future potential health impacts using scenarios of future changes in climate, socioeconomic, and other factors; *f*) synthesize the results; and *g*) identify additional adaptation policies and measures to reduce potential negative health impacts. Key issues for ensuring that an assessment is informative, timely, and useful include stakeholder involvement, an adequate management structure, and a communication strategy.

Over the past decade, it has become clear that the world’s climate is changing. In 2001 the Intergovernmental Panel on Climate Change concluded that there is new and stronger evidence that most of the warming observed over the past 50 years is attributable to human activities (Albritton and Meiro Filho 2001). The Intergovernmental Panel on Climate Change projected that the global mean temperature of the earth would increase by the end of the 21st century by between 1.4 and 5.8°C. Global precipitation patterns will also change. This projected rate of warming is much faster than the observed changes during the 20th century and is very likely to be without precedent during at least the last 10,000 years (Albritton and Meiro Filho 2001).

The primary international response to control greenhouse gas emissions is the Kyoto Protocol negotiated under the United Nations Framework Convention on Climate Change (UNFCCC 2005). The text of the protocol was adopted at the third session of the Conference of the Parties to the UNFCCC in Kyoto, Japan, on 11 December 1997 and entered into force on 16 February 2005. Because of the long lifetime of some greenhouse gases and the inherent inertia in the climate system, even full compliance with the Kyoto protocol means that adaptation to climate change will be required for at least several decades (Albritton and Meiro Filho 2001). Recent research and policy attention has therefore focused on assessments of potential vulnerabilities and identification of adaptation strategies, policies, and measures (Lim and Spanger-Siegfried 2004; Willows and Connell 2003).

Three broad categories of health impacts are associated with climatic conditions: impacts that are directly related to weather/climate, impacts that result from environmental changes that occur in response to climatic change, and impacts resulting from consequences of climate-induced economic dislocation, environmental decline, and conflict (McMichael et al. 2001). Changes in the frequency and intensity of heat events and extreme rainfall events (i.e., floods and droughts) will directly affect population health. Indirect impacts will occur through changes in the range and intensity of infectious diseases and food- and waterborne diseases, and changes in the prevalence of diseases associated with air pollutants and aeroallergens.

Concerns about climate change have led international agencies, nongovernmental and regional institutions, and national organizations to undertake vulnerability and adaptation assessments. Few national communications and other UNFCCC-related assessments in low-income countries and economies in transition have addressed climate change–related health impacts in any detail because of limited data availability and a lack of guidance on assessment methods (Kovats et al. 2003a). To provide such guidance, and in response to the need to develop a flexible approach for country-driven health assessments, we developed a framework using familiar methods to evaluate the impacts of climate variability and change, to identify vulnerable populations, and to assess adaptation policies and measures (Kovats et al. 2003b). The assessment process is stakeholder driven and, as such, is designed to focus on local environmental and health priorities.

In this article we first define vulnerability and adaptation in the context of climate change. We next describe the steps in a vulnerability and adaptation assessment. Third, we discuss issues related to the process of conducting an assessment. We then expand on using risk management to address climate change–related health risks. A starting point for many climate change assessments should be evaluation of how populations currently cope with climate variability, particularly weather extremes such as floods, droughts, and heat events, to indicate where additional interventions are needed. Improving the capacity to cope with current climate variability will likely increase resilience to climate change.

The remaining articles in this mini-monograph describe completed assessments in small island states (Ebi et al. 2006), Portugal (Casimiro et al. 2006), Cuba (Ortíz Bultó et al. 2006), and the indigenous populations in Canada (Furgal and Seguin 2006). In addition, Campbell-Lendrum and Woodruff (2006) describe methods that the World Health Organization (WHO) use to estimate the attributable burden of health determinants and outcomes due to climate change.

## Vulnerability and Adaptation

Assessing the potential health impacts of climate variability and change requires understanding both the vulnerability of a population and its capacity to respond to new conditions.

Human health vulnerability to climate can be defined as a function of *a*) sensitivity, which includes the extent to which health, or the natural or social systems on which health outcomes depend, are sensitive to changes in weather and climate (the exposure–response relationship) and the characteristics of the population, such as its demographic structure; *b*) the exposure to the climate-related hazard, including the character, magnitude, and rate of climate variation; and *c*) the adaptation measures and actions in place to reduce the burden of a specific adverse health outcome (the adaptation baseline), the effectiveness of which may influence the exposure–response relationship.

Adaptation includes the strategies, policies, and measures (hereafter referred to as adaptation options) undertaken now and in the future to reduce the burden of climate-sensitive health determinants and outcomes. Adaptation can be anticipatory (actions taken in advance of climate change) or responsive and can encompass both spontaneous responses to climate variability and change by affected individuals and planned responses by governments or other institutions (Smit et al. 2001). An example of a public health adaptation is an early warning system for heat events.

Table 1 provides definitions and examples of coping and adaptive capacity. Coping capacity encompasses the interventions that are feasible to implement today (in a specific population), and adaptive capacity encompasses the strategies and policies that have the potential to expand future coping capacity (Yohe and Ebi 2005). The primary goal of building adaptive capacity is to reduce future vulnerability to climate variability and change. Increasing the adaptive capacity of a population shares goals similar to those for sustainable development—increasing the ability of countries, communities, and individuals to effectively and efficiently cope with the challenges of climate change.

An adaptation assessment describes specific options that can be implemented to reduce current and future vulnerability as well as the resources needed (financial, technologic, and human capital) to implement them. The information generated from an adaptation assessment can be combined with a cost–benefit analysis or other decision support tool to inform priority setting by policy makers (e.g., Willows and Connell 2003).

## Steps in a Vulnerability and Adaptation Assessment

Assessment of vulnerability and adaptation uses concepts similar to those used in health impact assessments. Not all steps may be possible or desirable in a particular assessment, and the determination of which steps to be included depends on the objectives and resources available. Assessments can have different levels of in-depth analysis depending on the objectives, the interest of stakeholders, and the funding available.

### Determine the scope of the assessment

The first step is to specify the scope of the assessment in relation to the health issues of concern today and of potential risk in the future, the geographic region to be covered by the assessment, and the time period. The responsible national or regional health authority can identify the health outcomes to be included in collaboration with, when appropriate, *a*) the authorities responsible for the social security, environmental affairs, and meteorologic offices; *b*) the research community; and *c*) other stakeholders such as nongovernmental organizations, business, and the public.

### Describe current associations between disease outcomes and climate variability and change

This step involves describing the current burden and recent trends in the incidence and prevalence of climate-sensitive health determinants and outcomes of importance in the population of interest and the reported associations between weather/climate and the health outcomes of concern. The associations may be based on routine statistics collected by national agencies or on published literature. Meteorologists can provide input into how to define and describe the important types of weather exposure, for example, the severity and frequency of extreme weather events. Adverse health outcomes associated with interannual climate variability, such as El Niño events, also can be considered (Kovats et al. 2003c). When possible, it is useful for decision makers to calculate the proportion of a disease burden that is attributable to weather and/or climate, such as what proportion of all cardiovascular deaths are attributable to high or low temperature or the number of deaths caused by floods.

If resources are available and data are of sufficient quality and quantity, then new epidemiologic analyses may be undertaken, taking into account modifying and/or interacting factors. For example, morbidity and mortality increase during periods with both extreme heat and high levels of air pollutants (O’Neill et al. 2003).

### Identify and describe current strategies, policies, and measures designed to reduce the burden of climate-sensitive health determinants and outcomes

The key questions to address for a specific health outcome include the following: *a*) What is being done now to reduce the burden of disease? How effective are these policies and measures? *b*) What can be done now to reduce current vulnerability? What are the main barriers to implementation (e.g., technology or political will)? *c*) What options should begin to be implemented to increase the range of possible future interventions?

For each health outcome, the activities and measures that institutions, communities, and individuals currently undertake to reduce the burden of disease can be identified from *a*) review of the literature; *b*) information available from international and regional agencies (WHO, the Pan American Health Organization, United Nations Environment Programme, and others) and national health and social welfare authorities (ministries of health); and *c*) consultations with other agencies and experts that deal with the impacts of the health outcome of concern (e.g., the agencies that deal with the weather disasters). For example, is an early warning system for heat events in place? If so, what activities are instituted during a heat event to reduce morbidity and mortality?

Ideally, the effectiveness of adaptation measures should be evaluated. An evaluation should consider approaches to monitor how the performance of a measure may change over time compared with the baseline. For example, if an early warning system for heat events is in place, evaluation can determine whether mortality during an event is lower with the system (Ebi et al. 2004).

Information generated from this assessment of the adaptation baseline can identify policies and measures that could be implemented now to reduce vulnerability and increase future adaptive capacity. Consideration needs to be given to who will implement new measures and the possible barriers that may be encountered.

### Review the health implications of the potential impacts of climate variability and change on other sectors

Assessments should be integrated across relevant sectors, especially water resources, agriculture, flood hazard management, and the built environment. The results of other assessments should be included to better understand issues such as the health implications of the direct impacts of climate change on the food supply and the risk of disasters (e.g., coastal or river flooding). In addition the impacts of implemented adaptation options in response to actual or projected climate change need to be evaluated in terms of potential adverse health effects. For example, recommending domestic water storage may have implications for vector breeding and the transmission of dengue. Because of the many possible interactions and types of feedback among sectors, development and other projects should be subject to environmental and health impact assessments.

### Estimate the future potential health impacts

Assessing future health impacts requires using climate and socioeconomic scenarios. The scenarios used can be assumptions about a certain amount of increase in global mean surface temperature (i.e., 1 or 2°C) or can be detailed quantitative scenarios. If available, national or regional downscaled climate scenarios should be used (Arnell et al. 2004; Hulme et al. 2002; Willows and Connell 2003). Similarly, the appropriate national- or local-level projections of population growth and aging should be used. Addressing potential impacts both in the near term (the next 20 years) and the long term (up to 2050 or 2080) is advisable because a near-term focus provides relevant information within the usual planning horizon of health agencies, and an estimate of impacts in the longer term is needed to develop a comprehensive adaptation strategy.

Estimation of the potential future health impacts of climate variability and change implies using an approach in which models of climate change (and other changes) drive climate–health associations (Campbell-Lendrum et al. 2006). Health models may be complex spatial models or based on a simple relationship between exposure and response. The use of climate scenario data has been addressed in detail elsewhere (Hulme et al. 2002; Nakicenovic and Swart 2000). Projections may be incorporated from models developed for other sectors, such as flood risk, food supply, and land-use changes.

Policy makers must understand the multiple sources of uncertainty in estimations of potential future impacts, from climate projections to the climate/health models. Uncertainties begin with the climate models themselves, due to such factors as the complexity of climate systems, the possibility of nonlinear responses to changing greenhouse gas concentrations, variations in assumptions/model input, and lack of resolution at the regional and national levels. In addition there are multiple sources of uncertainty in climate/health models, including a lack of understanding of the key determinants of the geographic range and intensity of climate-sensitive health determinants and outcomes (e.g., the role of land-use change in the spread of vectorborne diseases), incomplete data on these relationships, a lack of understanding of how to mathematically model the relationships to make projections of future burdens of disease, and how societies and burdens of disease will change over the next 25–100 years (McMichael et al. 2001). Explicitly estimating uncertainty can further understanding of the level of confidence in what is known and can provide input into future research directions and policy making (Moss and Schneider 2000). Policy makers should be realistic about the likelihood that the uncertainty can be resolved in a meaningful time frame.

### Synthesize the results

The quantitative and qualitative information collected in the previous steps is synthesized to identify changes in risk patterns and to identify links among sectors, vulnerable groups, and stakeholder responses. It is important that the synthesis focus both on long-term projections to identify emerging trends and on the shorter time frames used in decisionmaking. Examples of assessments that synthesized qualitative and quantitative data are presented in Casimiro et al. (2006) and Furgal and Sequin (2006). The key issues that need to be communicated to decision makers and stakeholders include the specific projected health impacts, the current and projected burden of those impacts, the effectiveness of current interventions to control the health impact, the rate at which negative impacts could be detected, and the degree of certainty associated with the projections. Qualitative results can be summarized as, for example, a particular health outcome increasing from a low to medium level of concern over the next few decades with a high degree of certainty, depending on the effectiveness of interventions implemented to reduced the disease burden. Convening an interdisciplinary panel of experts with relevant expertise is one approach to developing a consensus assessment. Once synthesized, the information should be peer reviewed and published.

Assumptions that underlie any quantitative estimates should be clearly described. Quantitative estimates should be clearly identified with its climate scenario. The degree of certainty of qualitative and quantitative statements should be provided, and the most vulnerable population groups should be identified.

Value judgments have to be made in summarizing the assessment. In particular, decisions should be made about how to balance near-term and long-term effects; weigh the potential effects in different population groups; balance the more certain, quantifiable potential effects with those that are less certain and not quantifiable; and balance the interests of the various stakeholder groups (Lehto and Ritsatakis, unpublished data).

### Identify additional adaptation policies and measures, including procedures for evaluation after implementation

This step identifies possible adaptation measures that could be undertaken over the short term to increase the capacity of individuals, communities, and countries to effectively cope with the weather or climate exposure of concern. A review of adaptation measures implemented in other regions with similar health concerns may be one source of new adaptations. These measures should be possible to implement within the population’s access to material resources, technology, and human and social capital. For example, if heat-related morbidity and mortality are health issues in an urban area and if an early warning system for heat waves has not been implemented, then would implementing such a system likely benefit population health? Strengths and weaknesses as well as opportunities and threats to implementation should be evaluated and priorities set.

In addition, countries need to adapt to long-term climate change. The second aim of this step is to identify possible measures that can be taken today and in the future to increase the ability of individuals, communities, and institutions to effectively cope with future weather, including extreme weather events. Consideration should be given to the lessons learned from past public health policies, including the effectiveness of various measures, such as vector control and early warning systems.

Many of the possible measures for adapting to climate change lie primarily outside the direct control of the health sector. They are rooted in areas such as sanitation and water supply, education, agriculture, trade, tourism, transport, development, and housing. Intersectoral and cross-sectoral adaptation strategies are needed to reduce the potential health impacts of climate change. A policy analysis will determine the feasibility of and priorities among these options. Generally, many of the policies and measures identified also promote sustainable development.

Criteria should be established in advance for evaluating possible adaptation measures. Evaluation should be an ongoing process both to identify opportunities for improving the effectiveness of the measures but also to identify maladaptation and unintended consequences as quickly as possible (Yohe and Ebi 2005). The traditional public health methods for evaluating the efficacy and effectiveness of a particular intervention should be applied, with appropriate consideration of the local circumstances. For example, the effectiveness of heat event early warning systems can be evaluated by determining whether mortality during heat events decreases after system implementation. This, of course, requires that evaluation criteria be built into the system when it is developed.

## Framework for the Assessment

For an assessment to be informative, timely, and useful, key issues need to be addressed, particularly stakeholder involvement, an adequate management structure, and a communication strategy.

Experiences from countries that have performed assessments have shown the importance of including stakeholders in assessment planning, implementation, and evaluation. Stakeholders include people within governments, nongovernmental organizations, research institutions, and private entities that focus on public health. The issues and questions of greatest concern to the stakeholders must be elicited to ensure that the assessment provides useful information. This does not imply that relevant issues, otherwise not identified or known as important to stakeholders, would be left out of the assessment. Assessors strive to answer stakeholder questions to the extent possible given uncertain science; they also characterize the uncertainty and explore the implication for various policy or resource management decisions. Once an assessment is completed and the stakeholders are informed of the results, assessors should elicit from the stakeholders any new interests and concerns the assessment raises. Openness and inclusiveness enable participants to bring a diversity of views and information that may benefit the assessment process and make the process more transparent and credible.

An adequate communication strategy is needed before, during, and after the assessment. Effective risk communications is a two-way process including exchanges among interested parties (individuals, social groups, industry, and governments) (National Research Council 1989). Risk communication is, by definition, proactive and may involve many stakeholders and audiences, various levels of communication, and phases or stages of communication to accommodate the needs inherent in each step of the assessment. The potential for achieving successful risk communication increases with knowledge of the audience—what their concerns are, how they perceive risk, and whom they trust. Identifying this information early and incorporating it into the initial stages can bring benefits later in the process.

Many of the aspects of the assessment process, such as engaging stakeholders, synthesizing results, and developing policy, will have implications after the assessment is completed. Research gaps and information needs identified during the assessment will establish directions for future development. Selecting and implementing policy options feed into further monitoring and surveillance work to create an iterative cycle of assessment and policy development (Scheraga et al. 2003). For example, research gaps that are identified should guide the priority setting of research to fill these gaps, and new research findings can advance future assessments.

## Risk Management

Applying appropriate risk management principles, tools, and measures can reduce current and future human health vulnerability to climate variability and change. Numerous risk management frameworks have been developed that can be modified to address national, regional, and local assessment needs (e.g., Willows and Connell 2003). The first step in these frameworks typically involves evaluation of whether a specific exposure is a risk to human health and well-being. Once a type of exposure is determined to be a risk (e.g., heavy rain causing rivers to overflow), the consequences of exposure for the affected population are assessed, including the magnitude and frequency of the risk, the likelihood of exposure, who is or will be at increased risk of adverse health effects by level of exposure, and what is or will be at risk that could adversely affect health, such as damage to built infrastructure and/or interference with health and social services.

Risk identification is followed by an assessment of the strengths and weaknesses of the human and material resources available to reduce (or manage) the risks. This might include assessing the ability of public health units, fire departments, emergency services, and even military units to provide emergency services during weather-related disasters. There also should be an assessment of the ability to cope with risks that increase gradually, such as progressive droughts shrinking water supplies and increasing crop failures. Policy makers and the public need to know whether public health services and other health and social infrastructure might be weakened by a deteriorating economy and by shrinking government income and resources.

Next, information is needed on the awareness and tolerance of risk at the local, regional, and national levels. Information should be gathered on the risks that stakeholders perceive to be the most important and why. Priorities need to be established for how, by whom, how quickly, to what extent, and in which order the risks should and could be reduced.

The adaptation assessment will have identified a range of possible options that could be implemented to address the risks of concern. These interventions have varying degrees of effectiveness, ease of implementation, expected disadvantages, and costs. Decision makers and policy makers combine this information with factors such as current policy priorities and social values to determine a strategic direction. Stakeholders should be made aware of the human and financial resource trade-offs required for the recommended adaptation options, and the uncertainties associated with both the climate change–related health impacts and the effectiveness of the proposed approaches to mitigate those impacts.

Finally, mechanisms for monitoring and evaluation need to be established to determine whether the measures implemented have the desired effect and whether midcourse corrections are needed. Corrections may arise because of changes in social, economic, environmental, and technologic conditions over time. Significant changes may require initiating a new cycle of assessment and risk management to take these changes into account.

## Discussion

Addressing climate change–related health impacts has become more urgent with the realization that impacts are already occurring (Patz et al. 2005). Considerably more information is needed on the pathways by which weather can affect health, on the subgroups most vulnerable to climate-sensitive health determinants and outcomes, and on the implications of climate change for public health policy and practice. Continuing current approaches to risks posed by weather and climate runs the risk that potentially effective adaptation options may be unidentified, unimplemented, or implemented too late, resulting in preventable illnesses and deaths and increased costs.

Adaptation options to address climate change–related health impacts will aim to be cost-effective in terms of lives saved and illness avoided. The focus should be on win–win strategies to improve public health regardless of the changes in weather and climate. Adding adaptation measures into existing programs may not be costly. For example, integrated vector management programs could adjust some monitoring sites to determine if a vector or the disease it carries is expanding or contracting its range. In addition there are opportunities to adapt to multiple factors. For example, the existence of federal flood insurance in the United States provides an incentive for development in high-risk coastal areas (as strongly evidenced in the 2005 hurricane season), which increases the risk of injury and death to coastal populations (Scheraga et al. 2003). Elimination of federal flood insurance today would reduce the size of the coastal communities currently at risk (at a financial cost to individuals living in coastal communities) and at future risk due to rising sea levels. The decision of whether to adapt now or later should be based on a comparison of the present value of expected net benefits associated with acting sooner or later (Scheraga et al. 2003).

Initial national assessments and communications made clear that a major constraint to conducting a vulnerability and adaptation assessment is the lack of high-quality long-term data sets, particularly in most low-income countries and many economies in transition, to understand current relationships between weather and climate and health determinants and outcomes. However, ministries of health, nongovernmental organizations, other organizations, and researchers can qualitatively estimate current health burdens and how these burdens could change under different scenarios of changing temperature and precipitation. Another constraint in conducting assessments is the lack of experience with doing so, which is why the secretariat for the UNFCCC, the United Nations Development Programme, and other organizations are providing training on methods and tools to build national capacity for evaluating vulnerability to climate variability and change and for mainstreaming adaptation decisions into ongoing processes, such as sustainable development plans.

Assessments of the potential health impacts of climate variability and change are needed to inform the development of adaptation options in health and other sectors and to provide information on the impacts and the adaptation requirements to international policy processes. The assessment must make the problem and the potential impacts explicit and clear to policy makers and should help decision makers in choosing among adaptation options designed to reduce negative impacts.

## Figures and Tables

**Table 1:** Examples of current and future human health vulnerability and adaptation.

Definition	Current	Future
Vulnerability: the degree to which individuals and systems are susceptible to or unable to cope with the adverse effects of climate change.	Populations living in areas on the fringe of the current distribution of malaria are at risk for epidemics if the range of the *Anopheles* vector changes.	Whether these populations might be vulnerable in the future depends, in part, on the implementation of timely and effective prevention activities.
Adaptation baseline: the adaptation measures and actions in place in a region or community to reduce the burden of a particular health outcome.	The exposure–response relationship is influenced by the current prevention measures aimed at reducing the burden of a disease. For example, the number of older adults adversely affected by a heat event depends on the numbers who have access to and who use air conditioning during a heat event.	Increasing access to and use of air conditioning will reduce the percentage of older adults who could be adversely affected by future heat events.
Coping capacity: the adaptation options that could be implemented now. Specific adaptation plans arise from a region or community’s coping capacity.	Several cities in middle-latitude countries have the level of material resources, effective institutions, and quality of public health infrastructure to establish and maintain early warning systems for heat events. Until implemented, these systems are within a city’s coping capacity.	Over time, adaptation options can move from being possible to being implemented (i.e., being part of the adaptation baseline). For example, universal access to adequate quantities of safe water is not yet possible, although significant progress has been made.
Adaptive capacity: the general ability of institutions, systems, and individuals to adjust to potential harm, to take advantage of opportunities, or to cope with the consequences of climate variability and change.	Adaptive capacity is the theoretical ability of a region or community to respond to the threats and opportunities presented by climate change. It encompasses both coping capacity and the options that have the potential to expand future coping capacity.	Over time, it is hoped that regions and communities will increase their resilience to what the future climate brings.

Adapted from Kovats et al. (2003b).
